# Evaluation of the Long-Term Impact on Quality After the End of Pharmacist-Driven Warfarin Therapy Management in Patients With Poor Quality of Anticoagulation Therapy

**DOI:** 10.3389/fphar.2020.01056

**Published:** 2020-07-14

**Authors:** Leiliane Rodrigues Marcatto, Luciana Sacilotto, Letícia Camargo Tavares, Debora Stephanie Pereira Souza, Natália Olivetti, Celia Maria Cassaro Strunz, Francisco Carlos Costa Darrieux, Maurício Ibrahim Scanavacca, Jose Eduardo Krieger, Alexandre Costa Pereira, Paulo Caleb Junior Lima Santos

**Affiliations:** ^1^ Laboratory of Genetics and Molecular Cardiology, Instituto do Coracao, Hospital das Clinicas HCFMUSP, Faculdade de Medicina, Universidade de Sao Paulo, Sao Paulo, Brazil; ^2^ Arrhythmia Unit, Instituto do Coracao, Hospital das Clinicas HCFMUSP, Faculdade de Medicina, Universidade de Sao Paulo, Sao Paulo, Brazil; ^3^ Department of Pharmacology, Escola Paulista de Medicina, Universidade Federal de São Paulo, EPM-Unifesp, São Paulo, Brazil; ^4^ Clinical Laboratory, Instituto do Coracao, Hospital das Clinicas HCFMUSP, Faculdade de Medicina da Umiversidade de Sao Paulo, Sao Paulo, Brazil

**Keywords:** anticoagulation, warfarin, pharmacist, pharmaceutical care, time in the therapeutic range

## Abstract

**Background:**

Warfarin is the most common oral anticoagulant drug, especially in low-income and emerging countries, because of the high cost of direct oral anticoagulant (DOACs), or when warfarin is the only proven therapy (mechanical prosthetic valve and kidney dysfunction). The quality of warfarin therapy is directly associated with dose management. Evidence shows that pharmaceutical care achieves a better quality of therapy with warfarin. However, there are no studies showing this intervention in a specific patient group with poor quality of anticoagulation in a long period after the end of the follow-up by a pharmacist. Thus, the aim of this study was to evaluate whether the quality of warfarin therapy driven by a pharmacist remains stable in the long term after the end of follow up with a pharmacist, in AF patients with poor quality of anticoagulation.

**Methods:**

This is a prospective study, which evaluated about 2,620 patients and selected 262 patients with AF and poor quality of anticoagulation therapy with warfarin (TTR<50% - based on the last three values of international normalized ratio). Pharmacist-driven therapy management was performed up to 12 weeks. Data from patients were evaluated 1 year after the end of the follow-up with pharmacist.

**Results:**

Comparison between mean TTR after 12 weeks of pharmaceutical care (54.1%) and mean TTR one year after the end of the pharmaceutical care (56.5%; p=0.081) did not achieve statistical difference, demonstrating that the increment of quality due to intervention of 12 weeks was maintained for 1 year after intervention.

**Conclusion:**

The long-term impact of pharmaceutical care was beneficial for patients with AF and poor quality of warfarin anticoagulation. This design might be an important strategy to treat a subgroup of patients without proven effectiveness of warfarin.

## Introduction

Atrial fibrillation (AF) is one of the comorbidities highly associated with thromboembolic events and heart failure. Usually, oral anticoagulants are prescribed to prevent the occurrence of thromboembolic events in these patients. Despite the availability of direct oral anticoagulants (DOACs), warfarin is the only approved treatment in some groups, including valvular atrial fibrillation, kidney dysfunction and mechanical heart valves (Scanavacca and Darrieux, 2016). In addition, it is the most commonly used anticoagulant in low-income and emerging countries, mainly because the cost is significantly lower (Ageno et al., 2012; Burn and Pirmohamed, 2018). Therefore, it is important to study ways to improve the quality of warfarin therapy.

In order to ensure safe anticoagulation therapy with warfarin, which presents a very narrow therapeutic index, numerous drug interactions and large interpatient drug response variability (Nutescu et al., 2006; Tavares et al., 2018), a regular assessment of the anticoagulation level is required. This is done by the systematic monitoring of International Normalized Ratio (INR), in a way that the target therapeutic value range is between 2.0 and 3.0, as recommended for AF patients (Rosendaal et al., 1993; Kirchhof et al., 2016). The parameter that reflects the quality of warfarin anticoagulation therapy is the Time in the Therapeutic Range (TTR). Evidence has shown that higher values of TTR (>70%) are associated with higher warfarin effectiveness and fewer warfarin side events (Camm et al., 2012; Razouki et al., 2014), while values of TTR<50% are considered as poor anticoagulation control, presenting a higher risk of adverse events, both thromboembolic and hemorrhagic (Connolly et al., 2008).

Evidence shows that the quality of warfarin therapy is directly associated with dose management (Baker et al., 2009). In this context, a lot of attempts have been made to optimize the benefits of warfarin therapy. One of them is pharmacist-driven therapy management, which is based on the patient’s orientation and education with a follow-up by a pharmacist. Studies in the literature have assessed the benefit of pharmacist-driven therapy management in the general population (Hall et al., 2011). Therefore, in a previous study, we showed that AF patients with poor quality of anticoagulation significantly improved their TTR metrics after being followed-up by a clinical pharmacist for 12 weeks (14.4% ± 1.0 vs 54.3% ± 1.4, p<0.001) (Marcatto et al., 2018). However, there are no studies in the literature showing whether the quality of anticoagulation achieved by the patient during pharmacist-driven therapy management on an AF population with low TTR remains after the end of a follow up with a pharmacist. Thus, the aim of this study was to evaluate whether the quality of warfarin therapy driven by a pharmacist remains in the long term after the end of a follow up with a pharmacist in AF patients with poor quality of anticoagulation.

## Methods

### Setting and Study Design

This prospective study evaluated about 2,620 patients and selected 262 patients with AF and poor quality of anticoagulation therapy with warfarin over 18 years. Poor quality of anticoagulation therapy with warfarin was defined as a TTR<50%, based on the last three values of the International Normalized Ratio (INR) measured before the patient entered the study, considering a target therapeutic value range of between 2.0 and 3.0. To calculate TTR values, we accessed the medical records for previous INR values, independent of the periods, for all patients. Thus, TTR values were calculated by the Rosendaal method, which uses linear interpolation (Rosendaal et al., 1993). This cohort comes from the study published by our group in 2018. Patients were enrolled from January, 2016 to February, 2018, representing approximately 10% of the patients attended at the Arrhythmia Unit during the referred period (Marcatto et al., 2018). The protocol was approved by the Ethics Committee for Medical Research on Human Beings of the Heart Institute (InCor), Faculdade de Medicina FMUSP, Universidade de São Paulo (SDC 4033/14/013) and all participants signed the consent form.

We excluded patients with liver and/or kidney dysfunctions (AST- aspartate aminotransferase/ALT- alanine aminotransferase >3x normal; creatinine >2.26 mg/dl or >200 µmol/L or GFR<60 mL/min/1,73m^2^), alcoholism (≥8 drinks/week), valvular heart disease, use of another anticoagulant, chemotherapy treatment and patients who had changed their amiodarone dosage 1 week before entering the study (Marcatto et al., 2016a).

**Figure 1:**
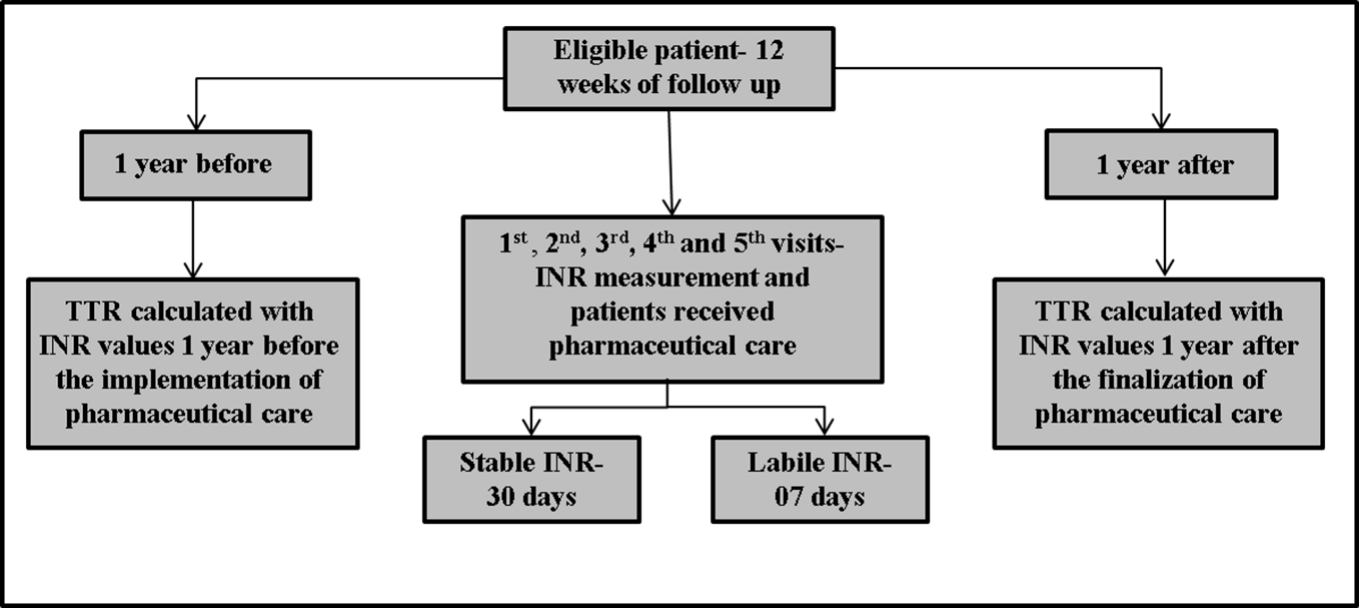
Study design. Patients were included in the study if they met the inclusion criteria and were followed-up for 12 weeks. The 1^st^, 2^nd^, 3^rd^, 4^th^, and 5^th^ visits occurred at intervals of 7 days each. On 5^th^ visit, if the International Normalized Ratio (INR) was stable (INR= ≥2.0 - ≤3.0), the patient returned in 30 days. However, if the INR was not stable (INR= <2.0 - >3.0), the patient returned in 7 days, until completing 12 weeks. At all visits, patients received pharmaceutical care, measured INR and if necessary, performed the dose adjustment. In addition, Time in the Therapeutic Range (TTR) was calculated 1 year before and 1 year after the follow-up by a clinical pharmacist.

**Figure 1** shows the study design. We evaluated these patients at different times: (1) 1 year before enrollment; (2) after follow-up with the pharmacist for 12 weeks (these first two points were previously published in 2018) (Marcatto et al., 2018); and (3) 1 year after the end of the follow-up with the pharmacist (i.e., after 12 weeks). The clinical laboratory calculated INR by the ratio PT (prothrombin time) of the patient divided by normal PT controls, elevated to the international sensitivity index. Past INR values and INR values from 1 year after the end of the follow up with pharmacist were checked in electronic medical records.

First, the medical team selected the patients according to the inclusion criteria. On the 1^st^ appointment, the study was explained to the patients by the pharmacist, and if the patient agreed to participate in the study, the consent form was signed. In addition, a pharmacist applied standardized questionnaires with personal, demographic, and clinical data. Afterwards, all data given by the patient were confirmed in the electronic medical records. We categorized the patients in self-declared racial/color parameter according to the Brazilian Census criteria. Following this parameter, we divided them into: White, Intermediate (meaning Brown, *Pardo* in Portuguese), or Black. We considered diabetes, dyslipidemia and those patients that were diagnosed as hypertensive before being included in the study. Additionally, cigarette smoking status, amiodarone use and use of inductor enzymatic drugs were categorized as a categorical variable (yes/no). Blood samples were collected from patients to measure creatinine, AST, ALT, and PT (prothrombin time) at a clinical laboratory to make sure that patients did not have kidney or liver disfunction.

During the follow-up, at every appointment, the patient’s INR was measured. If necessary, a dose adjustment was performed according to established guidelines (Ansell et al., 2008; Cushman et al., 2011). In addition, patients received pharmaceutical care at every appointment. Pharmaceutical care consisted of various actions: (i) providing the medication (we received the donation of Marevan^®^ 2.5mg, Marevan^®^ 5mg and Marevan^®^ 7.5mg from the Farmoquimica^©^ Pharmaceutical Industry); (ii) explaining all diseases and medications to patients, parents and caregivers; (iii) evaluating drug–drug and drug–food interactions; (iv) evaluating drug-related problems, adherence, and adverse events; and (iv) guiding and educating the patient about taking warfarin correctly. Adherence was checked by pill count; adverse events were checked by asking patients whether they had adverse events and by electronic medical records.

The 1^st^, 2^nd^, 3^rd^, 4^th^, and 5^th^ appointments occurred with an interval of 7 days each, regardless of the result of the INR test and if necessary, a dose adjustment. We considered patients to be dose stable when the patient’s INR value was within the therapeutic range (between 2.0 and 3.0). If, after the 5^th^ visit the patient had a stable INR, he or she returned after 30 days for a new measurement; if the patient had a labile INR (<1.8 or >3.2), the team performed the dose adjustment and the patient returned after 7 days for a new measurement. If the patient had an INR value of ≥ 1.8 and <2.0 or >3.0 and ≤ 3.2, warfarin dose was maintained and a new measurement was made in 7 days. Then, if the patient continued to demonstrate values of ≥1.8 and <2.0 or >3.0 and ≤ 3.2, warfarin dose was changed according to the guidelines (Ansell et al., 2008).

5Dose adjustment occurred according to the guidelines in the same way for all visits. In addition, we used the EP mobile tool for assisting in the management of the weekly dose (Horton and Bushwick, 1999; Ansell et al., 2008; Cushman et al., 2011): INR values ≤1.5, increased by 20%; >1.5 to < 2.0, increased by 5%; >3.0 to 3.5, decreased by 5%; >3.5 to <5.0 decreased by 20%; >5.0 to 9.0 decreased by 20% and omitted two doses and considered use of oral vitamin K; >9.0 discontinued warfarin for 7 days and considered administering vitamin K (Horton and Bushwick, 1999; Ansell et al., 2008; Cushman et al., 2011; Marcatto et al., 2016a).

Furthermore, TTR mean of one year before the pharmaceutical care and TTR mean of one year after the end of the pharmacist-driven therapy management were calculated for comparison with TTR mean after 12 weeks of pharmacist-driven therapy management. In both periods (before and after pharmacist-driven therapy management), patients received traditional warfarin therapy, i.e. the medical team provided drug information, evaluated adverse events and if necessary, performed warfarin dose adjustment. In these periods, the multidisciplinary team was not inserted. To calculate the TTR values for one year after the 12-week period and one year before, we used all INRs measured during this period. The number of times INR was measured varied from patient to patient, usually following the number of tests according to guidelines. If patients had an INR value within the therapeutic range, the INR was measured fewer times. Guidelines recommend measuring intervals of 30 to 90 days if the patient has an INR value within the therapeutic range. On the other hand, patients outside INR values had more INR measurements in a shorter time.

Information acquired during the study was managed using REDCap (Research Electronic Data Capture) tools hosted at Heart Institute (InCor), Faculdade de Medicina FMUSP, Universidade de São Paulo. REDCap is a secure, web-based application designed to support data capture for research studies (Harris et al., 2009).

### Outcomes

We evaluated the means of: (1) basal TTR (based on the last three INR values taken before the patient received the pharmacist-driven therapy management, with a cut-off for selection of TTR <50%). (2) TTR after 12 weeks (calculated based on the INR tests between 1^st^ and 13^th^ visits during pharmacist-driven therapy management). (3) TTR one year before and TTR one year after the end of the pharmacist-driven therapy management for patients with warfarin for anticoagulation. (4) TTR one year before and TTR one year after the end of the pharmacist-driven management were divided into four categories: (1) ≤ 30%; (2) > 30% to < 50%; (3) ≥ 50% to < 70%; (4) ≥ 70%.

### Statistical Analysis

We did descriptive analyses to present the clinical and demographic characteristics of the patients. Data are presented as mean and standard deviation (SD) or standard error (SE) of the mean. We used Kolmogorov-Smirnov and Shapiro-Wilk tests to check normality and TTR variable did not present a normal distribution. Therefore, we performed non-parametric test. We used Friedman’s two-way analysis of variance by ranks, for comparing the 3 TTRs. Friedman post-hoc Dunn test was used for pairwise comparisons. To compare the categorized TTR, Chi-square tests were performed. With a sample size of 262 patients, the study has a power of 100% to observe a difference of 20% between TTR means during different periods, using sigma of 25% and alpha of 0.05. With the same parameters and with a sample size of 99 patients, we were able to observe a difference of 10% between TTRs. Statistical analysis was carried out using SPSS software (v. 20.0, IBM SPSS, New York, NY, United States). The level of significance was set at p ≤ 0.05.

## Results

We included two hundred and sixty-two (n=262) patients and clinical and demographic characteristics are presented in **Table 1**. Patients had a mean age of 66.4 ± 11.7 years, 47.3% were female and predominant self-reported race/color was white (48.9%). The mean warfarin dose at inclusion was 28.5 ± 12.1 mg/week.

**Table 1:** Demographic and clinical characteristics of the patients (n=262).

Variable	Values
Gender (female), %	47.3
Age (years)	66.4 ± 11.7
Weight (kg)	78 ± 17
Height (cm)	165 ± 10
Self-reported race/color, % White Intermediate (Brown) Black Others*	48.9 38.2 11.5 1.4
Smoking, %	9.2
Amiodarone use, %	19.8
Use of inductor enzymatic drugs, %	1.1
Mean warfarin dose at time of inclusion (mg/week)	28.5 ± 12.1
Hypertension, %	81.7
Dyslipidemia, %	55.7
Diabetes, %	32.1
Time on warfarin medication (years)	5.2 ± 4.5

*Others: Amerindian or Asian. Continuous data are presented as mean ± SD.

**Figure 2:**
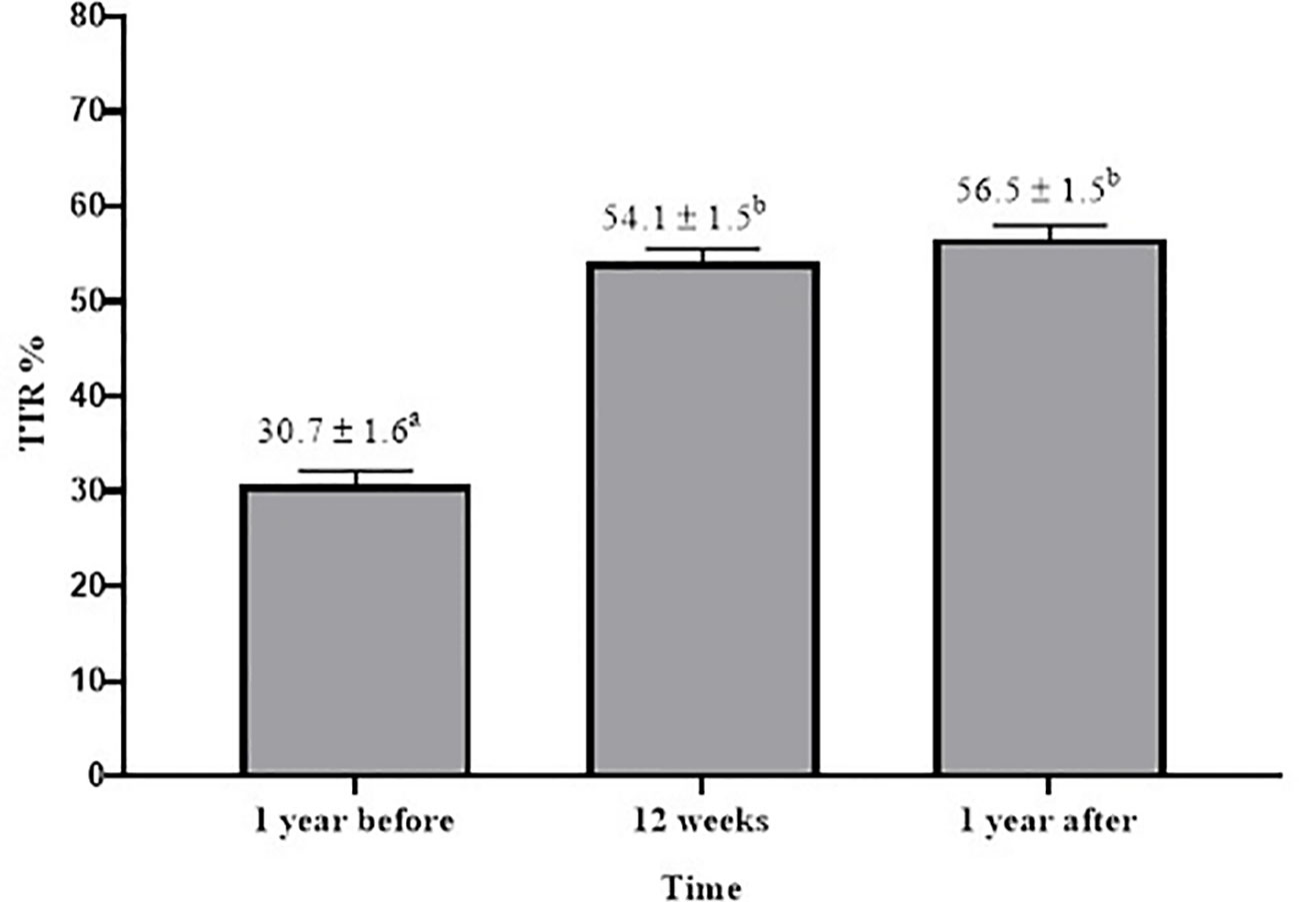
Comparison of Time in the Therapeutic Range (TTR) means in different periods (1 year before, after 12 weeks of pharmaceutical care and 1 year after). For this analysis, we used Friedman’s two-way analysis of variance by ranks, for comparing the 3 TTRs. Friedman post-hoc Dunn test was used for pairwise comparisons. Data are presented as mean % ± SE. Values with different superscript letters are significantly different (p < 0.001).

**Figure 2** shows that the mean TTR one year after the end of the pharmacist-driven therapy management was significantly higher than mean TTR one year before (56.5% vs. 30.7%, respectively; p<0.001). When we compared the mean TTR after 12 weeks of pharmacist-driven therapy management (54.1%) with mean TTR one year after the end of the follow-up, we did not observe a statistical difference (p=0.999).

**Figure 3:**
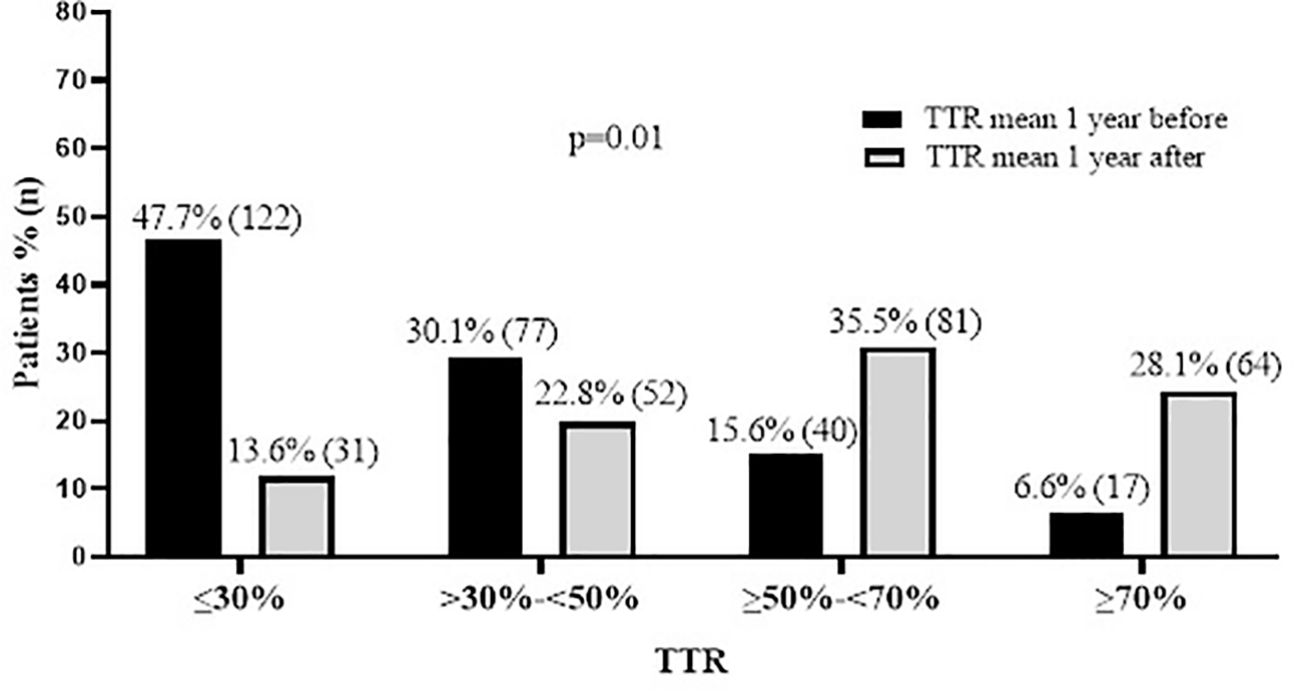
Comparison of percentages of patients that had Time in the Therapeutic Range (TTR)≤30%, TTR>30% - <50%, TTR ≥50%-<70%, and TTR ≥70% in different periods (1 year before and 1 year after pharmaceutical care). Data are presented as % (and absolute number of patients).

In addition, we divided patient groups into four categories according to mean TTR: (1) ≤ 30%; (2) > 30% to < 50%; (3) ≥ 50% to < 70%; (4) ≥ 70%. Thus, we identified that one year before the pharmacist-driven therapy management, 47.7% of the patients presented TTR ≤ 30% and only 6.6% of the patients presented TTR ≥ 70%. After 1 year of the pharmacist-driven therapy management, only 13.6% presented TTR ≤ 30% and 28.1% presented TTR ≥ 70% (p=0.01). We also observed that the majority of the patients (35.5%) presented TTR of ≥ 50% to < 70% one year after the end of the pharmacist-driven therapy management (**Figure 3**).

## Discussion

In this present prospective study, we used the TTR outcome one year after the end of the follow-up with the pharmacist (12 weeks) and we compared it with TTR one year before and the TTR after 12 weeks. Our findings show that pharmacist-driven therapy management for 12 weeks was effective and able to maintain the increment of quality, even one year after the end of the follow up with the pharmacist in patients with AF and poor quality of anticoagulation pharmacotherapy with warfarin.

To the best of our knowledge, there are no studies in the literature that evaluated the long-term impact of pharmaceutical care in patients with poor quality of anticoagulation pharmacotherapy with warfarin after the end of the follow up with a pharmacist. In addition, studies show that patients with poor quality of anticoagulation pharmacotherapy with warfarin with TTR<50% have a higher probability of adverse events, such as bleeding and thromboembolic events (Veeger et al., 2005; Connolly et al., 2008).

However, there are interesting studies, including general patients using warfarin. Mckenzie et al. (2018) showed that the long-term benefit of pharmaceutical care is maintained (after six months of pharmaceutical care), proving that this benefit is not only for patients with poor quality of anticoagulation therapy (TTR<50%), but also for general patients. They implied a TTR mean value of between 36.5% and 56.4% after 6 months. In a randomized study, Falamić et al. showed that after 6 months of follow up with the pharmacist, the mean TTR was significantly higher in the intervention group (93.0%) compared to the control (31.2%; p<0.001) (Falamić et al., 2018). However, they did not evaluate whether the quality of anticoagulation is maintained after the end of the follow up as we did.

Not all patients included in the study reached an optimal control of >70% (Camm et al., 2012; Razouki et al., 2014). However, the implementation of pharmaceutical care was able to increase TTR by about 25% when compared to the TTR mean one year before the pharmacist-driven therapy management and one year after the end of this service. Moreover, 28% of the patients reached a TTR value ≥ 70% one year after the end of the follow up with a pharmacist, but one year before the pharmaceutical care, only 6% reached a TTR value ≥ 70%. It is likely that all patients included in the study did not reach an optimal control of >70% because our sample is composed of patients with poor quality of anticoagulation with warfarin, which makes it harder to reach an optimal control. In addition, many factors were related to poor anticoagulation control, such as dietary habits, concomitant therapy, comorbidities, genetics and others (Santos et al., 2013; Marcatto et al., 2016b). “Although the literature shows that many factors can influence warfarin dose, we did not find these significant associations in our study, probably due to our specific type of patient cohort and selection methods. Furthermore, we believe that adherence is the main factor influencing TTR in our cohort. However, we did not have the data for patient adherence before entering the study or after the end of the follow-up with the pharmacist to prove this hypothesis

Another important outcome evaluated in several studies was the adverse event rate. Some studies have showed that the risk of hemorrhage and thrombosis events were significantly lower in pharmacist-led management groups (Hou et al., 2017; Falamić et al., 2018). In our study, we were not able to evaluate the adverse events, because our statistical power for this outcome is low and the present study is not a randomized trial, which would permit an adequate comparison between groups. However, in our study, there were no patients with major bleeding events or thromboembolic events. They only presented minor bleeding events (epistaxis, ecchymosis, hematuria, and menorrhagia).

Thus, the main finding of this study is that pharmaceutical care improves TTR and patients are able to maintain the acquired quality even 1 year after the end of pharmaceutical care. The data is important for developing and underdeveloped countries that do not have the financial support for a pharmaceutical service and/or other pharmacological classes of anticoagulants for all patients for a long period.

Our study has some limitations. First, it was not a randomized controlled trial, because patients are compared with themselves at different times. Second, we were unable to evaluate major adverse events because we followed up patients for only 12 weeks, and this period is considered insufficient to evaluate this type of outcome. Third, our study only evaluated patients with non-valvular AF. Consequently, the findings cannot be applied to groups of patients with other therapeutic indications for warfarin, for example, patients with heart valve disease. Fourth, we excluded patients with kidney or liver dysfunction, because the literature shows that these diseases have an important influence on warfarin dose and we decided evaluated only the impact of pharmaceutical care in modifiable variables like adherence. However, it is important to conduct a study in these patients. However, few patients were excluded because of kidney or liver disease. Most patients were excluded because they had TTR>50%”.

## Conclusion

In conclusion, the long-term impact of the pharmaceutical care was beneficial for patients with AF and poor quality of warfarin anticoagulation even after the end of follow up with the pharmacist. The design to identify and manage this part of a specific patient group might be an important strategy for countries and real-life settings that do not have access or resources to select DOACs to treat a subgroup of patients for whom warfarin has been ineffective.

## Data Availability Statement

The raw data supporting the conclusions of this article will be made available by the authors, without undue reservation, to any qualified researcher.

## Ethics Statement

This study involving human participants was reviewed and approved by the Ethics Committee for Medical Research on Human Beings of the Heart Institute (InCor), Faculdade de Medicina FMUSP, Universidade de São Paulo, approved the protocol (SDC 4033/14/013). The patients/participants provided their written informed consent to participate in this study.

## Author Contributions

LM followed up the patients, collected, and analyzed the data, and wrote the paper. LT analyzed the data and wrote the paper. DS collected the data. LS, FD, MS, and NO recruited the patients and critically revised the manuscript. CS, JK, and AP provided the facilities and critically revised the manuscript. PS analyzed the data and critically revised the manuscript. All authors contributed to the article and approved the submitted version.

## Funding

The São Paulo Research Foundation (FAPESP) funded this study (grant numbers: #2013/09295-3, #2016/22507-8, #2016/23454-5 and 2019/08338-7). Farmoquimica^©^ Pharmaceutical Industry donated Marevan^®^ 2.5mg, Marevan^®^ 5mg and Marevan^®^ 7.5mg.

## Conflict of Interest

The authors declare that the research was conducted in the absence of any commercial or financial relationships that could be construed as a potential conflict of interest.
